# The Changing Paradigm in the Management of Spinal Deformities

**DOI:** 10.2174/1874325001711011449

**Published:** 2017-12-29

**Authors:** Hans-Rudolf Weiss, Marc Moramarco

**Affiliations:** Gesundheitsforum Nahetal, Alzeyer Str. 23, D-55457 Gensingen, Germany; Scoliosis 3DC, 3 Baldwin Green Common, Woburn, MA, 01801, USA

Deformities of the spine and trunk are a cause of great anxiety to patients and their parents. While cosmetic appearance is a prevalent concern, spinal deformities rarely lead to severe health issues or affect life span. While thousands of patients undergo spinal fusion surgery annually, it is difficult not to question the necessity for surgery when patients–even those with fairly severe curves–rarely present with complications, cardio-pulmonary compromise or impactful symptoms (aside from the cosmetic). It is for this reason that the benefits and the risks of surgery be considered carefully. Active management, in terms of the highest quality conservative treatments and pattern-specific rehabilitation and/or bracing, should play a role. It is the most prudent path. For exercise instruction, patients must be taught the most up-to-date methods and acquire techniques they are able to use independently, without having to rely on a therapist. As a result of this training, the patient gains an understanding of their spine and protocols to be integrated daily, for the long-term when necessary. These methods, both for scoliosis and hyperkyphosis, are under continual development.

For thoracic kyphosis, the normal Cobb angle range is 25°-45° [1]. When Cobb angle exceeds 45°, it is considered to be an excessive outward curvature of the thoracic spine and is termed hyperkyphosis. Bracing is typically recommended when the Cobb angle, measured on a lateral spinal x-ray, reaches 55°-70°. Surgery is typically recommended when the Cobb angle is 70° or more [1]. Hyperkyphosis in adolescents mainly appears in conjunction with the typical signs of Scheuermann’s disease and rarely occurs as a result of vertebral malformation [1].

Scoliosis, on the other hand, is the result of numerous potential causes [2]. The term scoliosis describes a complex three-dimensional deformation of the spine and trunk. More specifically, scoliosis is a lateral curvature of the spine greater than a 10° Cobb angle (measured on an AP/PA spinal x-ray) and is usually accompanied by vertebral rotation. 80 – 90% of scoliosis cases are considered to be idiopathic [3]. Late-onset idiopathic scoliosis is the most common type and is also commonly referred to as adolescent idiopathic scoliosis (AIS) [3]. According to Weinstein et al. in their 50-year natural history study [4], AIS almost never leads to life-threatening consequences. Complications as result of scoliosis, such as dyspnea and/or right cardiac strain, do not typically occur until Cobb angles reach or exceed extremely severe measurements [3].

For progressive scoliosis and hyperkyphosis, the traditional treatment approaches have been bracing and spinal fusion surgery. Bracing has been controversial for years, but a 2013 study on bracing for scoliosis (BrAIST) [5] has put most criticisms to rest, at least for scoliosis curves up to 40º, the upper parameter for patients included in that study.

For AIS, spinal fusion surgery is recommended for growing adolescents at various Cobb angles, but typically around 45º to 50º. Some surgeons operate on curves as low as 40º. Despite this, no long-term study provides Level I evidence in favor of surgery when compared to the natural history of scoliosis referenced above [6-8]. Furthermore, a post-surgical study with a five to twenty-year follow-up demonstrates fairly high complication rates of 25% to 40% and more [9]. According to Hawes, those complications include pain, late infection and the need for re-surgery [6].

Recently, conservative approaches are used increasingly and gaining acceptance. With that said, there are diverse conservative approaches and practitioners of varied experience. Many approaches lack a long-term history or evidence. The few available RCTs support the Schroth method and/or treatment components such as active self-correction which is included in the current treatment protocols of the method’s newest evolution, Schroth Best Practice^®^ [10-12]. As mentioned previously, BrAIST [5] provides evidence in support of rigid bracing, primarily using the symmetric Boston brace. Some Level II evidence also exists in support of Chêneau-style bracing [13]. The Gensingen brace, a Schroth compatible, 3D-concept Chêneau brace, takes a patient’s individual spinal curve pattern into account to produce an asymmetric scoliosis brace which strives to provide the optimal in-brace spinal corrections [14]. Further research to compare the efficacy of the various brace types is needed. Another important factor is patient’s comfort and quality of life since a brace for scoliosis or hyperkyphosis usually needs to be worn for a significant number of hours each day. What is imperative to understand for both the patient and the practitioner is that when it comes to the conservative management of spinal deformities, whether it is via exercise, bracing, or both, there is a clear correlation between corrective effect, patient compliance, and treatment outcome.

In this issue, we will describe and show evidence in support of current non-surgical practices, all of which aim to stop the progression of spinal deformities, but ideally attempt some degree of correction in an adolescent prior to skeletal maturity. It is important to highlight the efficacy of these less invasive options so that families may pursue the most advantageous treatment strategy, in a timely manner, for their child with scoliosis or hyperkyphosis.

This issue will include a review of the most recent evidence for the treatment of spinal deformities in adolescents and adults, a comprehensive review of the etiological theories of idiopathic scoliosis, as well as a historical overview of Schroth conservative therapies. Other topics of interest are: an overview of the imaging methods used to diagnose and monitor patients with spinal deformities, the role of correction in conservative treatments, an intermediate prospective cohort study of scoliosis patients with Cobb angles of ≥40° treated with the Gensingen brace, and a case study of a nearly-skeletally mature patient treated with pattern-specific scoliosis rehabilitation (PSSR).

Typically, exercise-based programs for scoliosis are classified as PSSE (physiotherapeutic scoliosis-specific exercises), however, in this issue, we will describe the comprehensive Schroth Best Practice^®^ program of instruction which we differentiate with the acronym PSSR.

Thank you to all the authors for their dedication to the field and their tremendous efforts with this special issue. This issue would not have come to fruition without the hard work of Maja Fadzan who helped coordinate this project with the many authors from across the oceans and continents. We look forward to moving the conversation forward about the effective management of conservative therapies for the benefit of all patients afflicted with spinal deformities.
